# Financial Crisis Warning for Listed Manufacturing Companies in China

**DOI:** 10.1155/2022/1439057

**Published:** 2022-04-22

**Authors:** Yang Wang, Weimin Liu, Yuwei Fu

**Affiliations:** China University of Mining and Technology, Beijing 100083, China

## Abstract

In this paper, we have first selected 28 indicators based on the selection principle of financial indicators adopted in relevant studies both at home and abroad from seven aspects, which include profitability, long-term and short-term solvency, company development capacity, operating capacity, cash flow generation capability, equity characteristics, and board characteristics. Subsequently, we have conducted the comparative analysis and comprehensive study on the logistic early warning model and BP neural network to provide reference for managers and stakeholders to select the optimal model. In addition, through our study on the dynamic early warning of BP neural network, we intend to convey the concept of constantly updating the model to both managers and stakeholders. Therefore, this paper provides ideas for the research on the model of financial crisis early warning for China's manufacturing industry. The study is of significance for guiding the research of related issues in the manufacturing sector and can also provide reference for the early warning of other industries.

## 1. Introduction

Overseas scholars have made the earliest attempt to study the early warning model and have obtained fruitful results out of their research. In contrast, domestic scholars started their research in this respect rather late and the model established accordingly is not mature yet. Fitzpatrick was the first researcher to propose the use of single variable early warning model and finally concluded that the equity ratio and the return of equity (ROE) could play a significant role in identifying enterprises mired in financial crisis [1]. Altman was the first scholar to conduct the multivariate linear analysis, and he set up a Z-score model to forecast the financial status of an enterprise by using the equity ratio, ratio of working capital to total assets, ratio of EBIT to total assets, total asset turnover, and ratio of retained earnings to total assets [2]. Wu et al. took into account the profit-based risk management while setting up a multiple logistic regression model with traditional financial indicators and verified the significance of the early warning model. The multiple logistic model helps relax several constraints and enhance the accuracy, but it is prone to the issue of multicollinearity [3, 4]. Sarlin proposed to integrate the genetic algorithm with the neural network algorithm to optimize the latter one, thereby providing warning on the financial crisis [5]. Wu and Huang applied the models developed by overseas scholars in China for the first time and found that these models proved to have higher capacity of early warning for the enterprise crisis after univariate and multivariate analysis [6]. Based on the Z-score model developed by overseas scholars, Yang and Xu put forward the Y-score model, which is more consistent with China's national conditions as evidenced by its accuracy of about 90% [7]. Xu substituted the index variables after the principal components into the logistic model for early warning, which empowered the early warning model to become more effective [8]. Li and Tang adopted three different training functions to improve the BP algorithm, and they finally showed that the LM-BP algorithm is the optimal algorithm [9].

Judging from our comprehensive review of the methodology adopted in early warning models both at home and abroad, we may conclude that most of them adopt traditional statistical methods. Although in recent years, scholars are committed to introducing the BP neural network into financial early warning models, few scholars have established models on traditional statistical methods and BP neural network. Therefore, we have adopted the traditional statistical method and BP neural network to set up models, respectively, in this paper, and we have conducted the comparative analysis and comprehensive study of the models so as to provide the optimal forecasting method for the manufacturing industry.

Financial early warning indicators have undergone an evolution from traditional financial indicators to those containing cash flow indicators. The indicators selected in the first domestic financial early warning research also mainly rely on the basic financial indicators obtained from various financial data of enterprises. Chen and Zhang used the three indicators of asset-liability ratio, working capital to total asset ratio, and total asset profit rate in the index research of the model, indicating that these three indicators can achieve better warning effect [10, 11]. With the increasing emphasis on cash flow, cash flow indicators are constantly being introduced into domestic models. Zhang selected cash-to-liability ratio, sales cash ratio, asset cash recovery rate, etc. in the selection of indicators [12]. Deng and Du selected indicators such as operating leverage coefficient, company capital recovery rate, and asset recovery rate [13].

Many scholars believe that enterprises are in an open environment rather than a paradise, and non-financial indicators should be included in the research. Chen et al. incorporated EVA in the financial early warning model, an indicator that contains market information and can fully reflect the profitability of enterprises [14, 15]. Cao introduced corporate governance indicators such as investor protection indicators, related party transaction indicators, and shareholding ratio of major shareholders [16]. Zhang et al. believed that different industries have different risks and considered the industry environmental risk index [17, 18]. Cai introduced corporate governance indicators and macroeconomic indicators into the company's early warning model according to the company's internal and external environment to help companies find the factors that affect financial crisis [19]. Fang et al. believe that the financial status of enterprises in different periods is different; therefore, life cycle indicators are introduced into the early warning model [20]. Xu added corporate governance indicators on the basis of traditional financial indicators, which improved the accuracy of the early warning model [21].

## 2. Selection of Research Samples and Indicator Variables

### 2.1. Selection of Research Samples

Based on the available data, we selected 102 enterprises that encountered financial crisis and 102 enterprises with healthy financial situation in a ratio of 1 : 1 in the principle of the same industry, time periods, and similar total assets. Subsequently, we divided the selected samples into the samples for modeling and the ones for testing. Through the ransom extraction of SPSS, 60% of the total samples are selected as modeling samples and 40% are selected as testing samples. The final modeling sample amounts to 61 enterprises that encountered financial crisis and 61 matched enterprises with financial health, whereas the remaining 41 groups of enterprises are regarded as testing samples.

### 2.2. Selection of Indicator Variables

Based on the existing studies of indicators at home and abroad, the characteristics of the manufacturing industry, and China's current national conditions, we preliminarily selected 28 financial indicators that could reflect enterprises' financial status in this paper from seven aspects, namely, long-term and short-term solvency, company development capacity, operating capacity, profitability, cash flow generation capabilities, equity characteristics, and board characteristics, and the values of these indicators are directly extracted from the CSMAR database.

To establish an effective financial early warning model, we need to ensure that the selected indicators are able to effectively distinguish the companies mired in financial crisis from those with financial health. Therefore, subsequent to the preliminary selection of indicators, we need to further examine these indicators so as to select the ones that have significant identifying effect on the company's condition, which will enhance the effectiveness of the model to be established. In this paper, we used the software SPSS 19.0 to substitute all initial financial indicators into the KS testing model to determine whether they conform to the normal distribution. Data that conform to the normal distribution are set aside and substituted into the independent sample *T*-test model to determine whether they are significant, and those indicators that do not conform to the normal distribution are substituted into the non-parametric test model to determine whether they are significant. Last but not least, the significant indicators in independent sample *T*-test and non-parametric test are collected to establish the financial crisis early warning index system. Subsequent to the above selection procedures, the final early warning indicators are listed in Table 1.

## 3. Establishment and Empirical Test of the Financial Early Warning Model

### 3.1. Logistic-Based Warning Model

The logistic model is a function that studies the probability of occurrence of an event. It creates an expression function that reflects the probability of a crisis by studying the relationship between the independent variable and the response variable. The independent variable refers to the index data that need to be substituted into the model, and the dependent variable is the probability corresponding to the two firm states. By substituting the independent variable and response variable 1 and 0 into the model, the probability expression about the function of the independent variable can be obtained. The maximum likelihood estimation method used in this model is used to estimate the value of the parameter in the corresponding expression.

Berry and Feldman believed that it is necessary to reduce the multicollinearity between indicators in order to make the expression coefficient of logistic model accurately express the relationship between the indicator and the probability because reducing the multicollinearity between indexes can reduce the error of the logistic regression model [22–24]. In this paper, the principal component analysis is used to reduce the multicollinearity of the early warning indicator system finally formed in Table 2.

The logistic regression model is one of the methods of early warning adopted in this paper. However, after consulting the relevant literature, we found that the model has some limitations and that the collinearity of the indicators used in the model will affect its effectiveness in providing early warnings.

We used the method of the stepwise forward regression in SPSS19.0 to define ST companies as 1 and non-ST companies as 0. Whether the company becomes ST is the dependent variable, and the principal component after the principal component analysis (PCA) is set as the input variable of the logistic regression model. We can obtain the logistic regression expressions in the years of t-2 and t-3 from the parameter table of the logistic regression model accordingly.

According to Table 2, we can obtain the financial crisis early warning model for manufacturing enterprises, which is a discriminant of logistic regression shown as follows:

(1) $$\begin{array}{c} P=\frac{1}{1+e^{-\left(0.12-1.265\text{F}1-1.318\text{F}2-2.678\text{F}3-0.858\text{F}4-1.349\text{F}6-1.033\text{F}7\right)}}. \end{array}$$

We used the *P* value in the logistic regression expression to determine whether a company has encountered financial crisis. We set the value of 0.5 as the cutoff point, and when the value of *P* is [0.5, 1], the company is considered to experience a financial crisis. By comparison, when the value of *P* is [0, 0.5], the company is considered to be financially healthy.

Judging from Table 2 and the logistic regression expression, six principal components have passed the significance test at the confidence level of 0.05, which are F1 (cash flow generation capability), F2 (overall solvency), F3 (overall profitability), F4 (operating capacity), F6 (profitability in business), and F7 (equity characteristics).

According to column B of the table of variable parameters, the absolute value of coefficient in F3 is large and the coefficient term is negative, indicating that the improved overall profitability of enterprises can reduce the probability of financial crisis. Furthermore, the coefficients of F1, F2, and F6 are also negative, indicating that the improved cash profitability of enterprises can enhance the company's solvency and its ability to translate the main business into profits, so as to reduce the probability of financial crisis. In addition, the coefficients of F7 and F4 are negative, but with smaller absolute values compared with other indicators, indicating that the properly improved ability from these two aspects can also reduce the possibility of financial crisis.

According to Table 3, we can obtain the financial crisis early warning model for manufacturing enterprises, which is a discriminant of logistic regression shown as follows:

(2) $$\begin{array}{c} P=\frac{1}{1+e^{-\left(0.781-2.509\text{F}1+18.025\text{F}4-1.145\text{F}5-6.091\text{F}9\right)}}. \end{array}$$

Table 3 shows that four principal components have passed the significance tests at a confidence level of 0.05, which are F1 (overall solvency), F4 (growth capacity), F5 (profitability in business), and F9 (long-term solvency).

According to column B of the table of variable parameters, the coefficients of F1, F5, and F9 are negative, indicating that by improving the long-term and short-term solvency and increasing the profitability in terms of business income, the enterprises can reduce the probability of encountering the financial crisis. F4 is a positive coefficient representing the growth capacity of the enterprise. Based on the relationship between the coefficient and the financial crisis, the positive coefficient indicates that F4 is proportional to the financial crisis. In other words, by increasing the growth capacity, the company risks increasing the probability of financial crisis, which seems to run contrary with the common sense. Judging from the corresponding financial indicators of F4, it is found that the indicators representing the growth capacity of enterprises mainly include the growth rate of operating income and the growth rate of the owners' equity. By comparing the values of the two indicators in the financial crisis sample and the financially normal sample in the training sample set, we have identified the phenomenon that the growth rate of operating income or the growth rate of the owners' equity of the financial crisis companies exceeds 1, such as the companies with the stock codes 600228, 600074, 000408, 600234, 002289, and 000068. By contrast, the growth rates of operating income and owners' equity are less than 1 in almost all normal companies. By selecting the values of the years before and after the sample growth rate of several special companies, we have found that the growth rate of business income or the growth rate of the owners' equity in the research sample is situated at the inflection point in the data of the last three years. By contrast, the year selected for the indicators coincides with the year when the listed company got rid of ST last time, which makes the company's development ability directly proportional to the occurrence of financial crisis. Detailed statistics are shown in Table 4. The results indicate that the enterprise should maintain stability during its development since excessive development will also cause the enterprise to be mired in crisis once again.

The logistic regression model is established based on the above model. The accuracies of the training and test prediction in the years of t-2 and t-3 are shown in Tables 5 and 6. The results have shown that the accuracies of training and test samples in the year of t-2 are greater than those in the year of t-3, respectively. It shows that the closer the year of the financial crisis, the higher the accuracy of the prediction.

### 3.2. Early Warning Model Based on BP Neural Network

Artificial neural network (ANN) is a model for processing complex data. This model is a simulation of the function and structure of biological neurons because neurons are often connected to each other to form different structures but can operate normally. The neural network established by biological neurons is also composed of many different neurons. The weights are connected between layers. Once the structure is changed by external force, the neural network itself will pass the weights. Changes are made to accommodate such changes, thus again reaching the optimal model.

**Figure 1:**
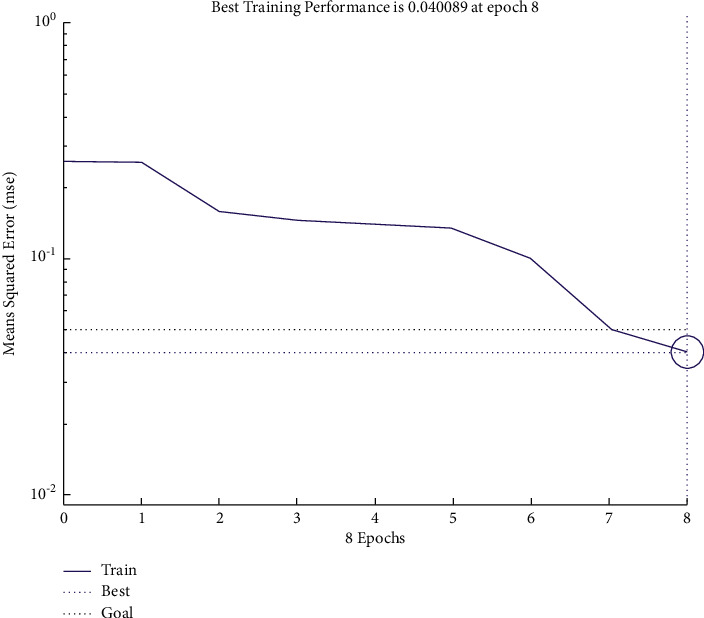
Error curve of the financial crisis early warning model in the year of t-2.

**Figure 2:**
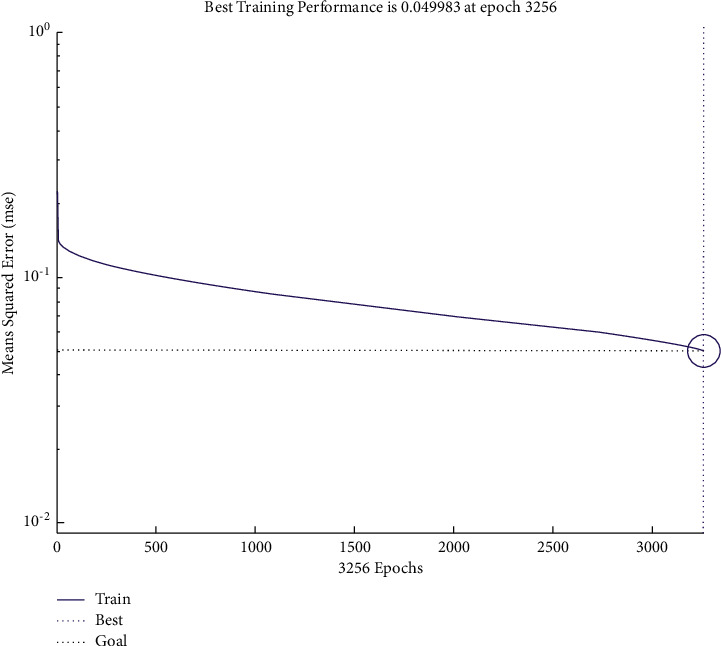
Error curve of the financial crisis early warning model in the year of t-3.

The number of input layers is 1, whereas the number of nodes in the input layer in the year of t-2 amounts to 23, which is the number of indicators finally selected. Furthermore, the number of nodes in the input layer in the year of t-3 is 21, which is also the number of indicators finally selected. We have set the number of layers and nodes of the output layer to 1. In addition, we have used the logsig function as a function between the hidden layer and the output layer and the tansig function as a transfer function from the input layer to the hidden layer. We have used the newff function to establish the network, and we have opted for the trainlm function to train the network. After repeated tests, we have set the parameters in this paper as follows: epochs are 5,000, goal is 0.05, and lr is 0.005; the number of hidden layer nodes in the year of t-2 year is set as 14, and the number of hidden layer nodes in the year of t-3 years is set as 12. The training performance in the years of t-2 and t-3 is shown in Figures 1 and 2.

The accuracies of the training and testing samples that we have obtained from BP neural network in the years of t-2 and t-3 are shown in Tables 7 and 8.  Table 8 shows that the accuracies in the years of t-2 and t-3 amount to 92.68% and 79.27%, respectively, indicating that the closer the year of the financial crisis, the more accurate the forecasts are.

### 3.3. Comparison and Integration of Financial Crisis Warning Models

#### 3.3.1. Comparison of Prediction Accuracy

A summary of the accuracies of the models based on the logistic regression and the BP network is shown in Tables 9 and 10.

By comparing the two models, we have found that the predictive accuracy of BP network is higher than that of the logistic regression method in both years of t-2 and t-3. By comparing the accuracies in the years of t-2 and t-3, we have found that the accuracy in the year of t-2 is generally higher than that in the year of t-3 for both the logistic method and the BP network method, indicating that the closer the year of the financial crisis, the more accurate the forecasts are.

#### 3.3.2. Comparison of Applicability of Methods

In terms of the selection of independent variables, we have found that a strong linear relationship between indicators in the logistic regression will reduce the prediction effectiveness of the model. BP network method has no restrictions in the selection of indicators, which can retain the indicators that may have an impact on the financial situation to the maximum extent. Even in case of fluctuations of data related to individual indicators data, there will not be a greater impact on the early warning effect of the model.

Logistic regression can reflect the relationship between the probability of a crisis and financial indicators by mathematical expression. Through the coefficient before the variables, we are able to clearly understand the positive and negative impact of variables on the crisis and the extent of such impact. However, due to the complex internal structure of the BP model, it is impossible to list clearly linear expressions between input and output. During the actual research, we can only resort to the output results to determine whether the model has reached a high level of prediction accuracy.

#### 3.3.3. Comprehensive Analysis on the Two Models

As the enterprise managers need to use the model to identify the relationship between the indicators and the financial status and to achieve a higher accuracy, they can jointly use the logistic regression and the BP network methods to find out which specific factors have a significant impact on the status of financial crisis through the logical regression expression. In this way, the management can adjust the company's business strategy during actual operation and get rid of the potential crisis.

Given that corporate investors and creditors are scattered and that investors may be small and medium-sized enterprises (SMEs) or individuals, coupled with the absence of a specialized mechanism for risk early warning, it is necessary to set up an early warning model to predict the financial condition of the company before investment and debt issuance. However, due to the tedious indicator processing during the early stage of the logistic model, it is recommended to use the BP model for early warning.

In view of the strong supervisory system and professional knowledge, the supervisory departments should leverage the high precision of BP to timely disclose to the public that listed companies may experience financial deterioration. In addition, regulators shall use the expression of the logistic model to remind enterprise managers of the problems they should pay attention to during operation.

## 4. Research on Dynamic Early Warning Based on BP Neural Network

In this section, we mainly intend to resolve the confusion that may still exist when enterprises actually select models for sample testing. Based on the training and testing in the years of t-2 and t-3 by the logistic model and the BP network model, respectively, in Section 2, we have taken the BP neural network as an example to illustrate whether we shall select the model with the span between data and status of two years or three years or with time interval from the test sample of one year or two years when testing the same test sample. Therefore, the three models of A, B, and C are established for verification in this paper, so that users can make more targeted selection of models according to the results of verification. Details of the three models are shown in Table 11.

The statistics for the three models are shown in Table 12. According to the training, testing, and forecasting by models A, B, and C, the accuracy of model A ˃ that of model B ˃ that of model C in predicting whether a financial crisis would take place in 2019. The results indicate that when selecting a model to predict the company status, there are two principles to follow for improving the accuracy. The first principle is the closest span between the data and the company status. In other words, model A should be selected among the three models since the span between model A and model B data and company status is 2 years, whereas that between model C is 3 years. The second principle is that the modeling time should be the closest to that of the test sample. In other words, model A should be selected in both A and B because the time interval between model A and the test sample is 1 year, whereas the time interval between model B and the test sample is 2 years. During the practical application of the company, the existing models should be updated if they are unable to meet the two principles. It should be noted that the span between data and company status in model C is 3 years. The test sample scenario is generally applicable for prediction when the financial data for the next two years are unknown. By contrast, if the data for the next two years are known, then model C is not recommended.

If an enterprise has already put in place an early warning model, both the updated and the previous models can then be used to forecast. Accordingly, the prediction results are analyzed based on the prediction of different models. If the predictions are consistent, the results would be quite reliable. If the predictions are inconsistent, a judgment can be made in combination with the internal situation of the company. For creditors and investors who may have no early warning models before, they should abide by the two principles when building new models to improve the accuracy of prediction (Table 12).

## 5. Conclusions

We have obtained the research variables and models that need to be used in this paper based on the research status of the financial crisis early warning variables and models at both home and abroad. During the selection of research variables, we have integrated the traditional financial indicators with cash flow indicators and non-financial indicators, and we have selected 28 indicators from seven aspects to carry out the KS test, independent sample *T* test, and non-parametric test on the initial financial indicators. In addition, we have selected the indicators of significance as the indicator system of the later study according to the test results. Logistic and BP network are combined during model selection. In the study of the logistic model, we have conducted the principal component analysis to reduce the dimension of the indicators, which are then substituted into the model, so as to identify the factors that affect the financial crisis based on the analysis of the logistic model. The analysis in the year of t-2 shows that by improving the cash flow generation capabilities, solvency, overall profitability, operational profitability, business profitability, and equity concentration, we can reduce the probability of financial crisis. Furthermore, the analysis in the year of t-3 shows that by improving overall solvency and operational profitability, in addition to long-term solvency, we are able to reduce the probability of financial crisis, and by increasing the development capacity, we are able to increase the probability of financial crisis. The accuracy of prediction in the year of t-2 is higher than that in the year of t-3 in the early warning by BP network. By integrating the two models, it is found that the prediction accuracy of BP neural network is higher than that of the logistic model, and the logistic model can be used to determine the factors affecting financial crisis instead of BP. According to the dynamic early warning model of BP neural network, the models should be constantly updated in the actual research to make it closest to the test sample year.

Therefore, this study is expected to provide theoretical support and guidance for both managers and other stakeholders to establish early warning models in the future and provide insights for other industry insiders to set up their corresponding models. Users of the financial early warning models are advised to take into account various early warning models based on their varying needs.

The following recommendations are made to business managers, investors and creditors, and regulators.

Firstly, for enterprise managers, it is necessary to enhance employees' awareness of early warning and improve the internal control system, so that in the process of daily work, once employees find signs of financial crisis, they will report them in time to avoid further deterioration of factors affecting financial crisis. In addition, in terms of early warning model establishment, since enterprises can learn the company's financial data in a timely manner, enterprises can update the early warning model in real time. When establishing the model, the early warning indicators studied in this paper can be used, or some new ones can be added according to the actual situation of the enterprise. For financial indicators, the logistic model and BP model can be used in combination, and a new model can be established according to the idea of constructing the model in this paper.

According to the logistic model established in this paper, the variables that have an impact on financial crisis mainly include profitability, solvency, operating ability, cash flow ability, equity characteristics, and development ability. Therefore, if an enterprise wants to improve its own profitability, it should attract talents, continuously learn advanced technologies at home and abroad, develop innovative products, enhance its core competitiveness, and seize domestic and foreign markets, thereby enhancing its own profitability. Regarding operating capacity, cash flow capacity, and solvency, the company should be as detailed as possible when formulating production and operation plans, not only taking into account the planning of its own production and operation activities but also taking into account the impact of financial risks, focusing on cash flow capacity indicators and debt repayment ability indicators, and grasping the balance between assets and liabilities. Cash flow should be reasonably controlled to avoid, as far as possible, financial crises caused by insufficient solvency or poor liquidity. For the characteristics of enterprise equity, the company can improve the corporate governance structure according to the actual situation and can conduct moderate concentration of equity, which can enhance the company's governance decision making. As for the development ability of the enterprise, it should not be overdeveloped for the temporary beauty of the report but should be developed steadily.

Secondly, for investors and creditors, since corporate investors and creditors are relatively scattered and investors may be small and medium-sized enterprises or individuals, there is no professional risk early warning mechanism, and it is impossible to obtain the financial data of enterprises in a timely manner to analyze. Therefore, investors and creditors can use the BP neural network early warning model constructed in this paper to predict the occurrence of corporate crisis because BP is more convenient to use than logistic regression. If investors and creditors find out that there is a financial crisis in the company through the model before investing and issuing bonds, then they should be cautious about investing and issuing bonds because the final principal may not be able to be recovered. If the model predicts that the financial state of the company is normal, it is also necessary to analyze the profitability and solvency of the company over the years before investing. If investors and creditors have invested in the company or issued bonds, the model should be updated regularly every year to further forecast the company, so that measures can be taken in a timely manner to prevent further losses.

Finally, for the regulatory authorities, since the samples of the model established in this paper use manufacturing A shares and the introduction of non-financial indicators is limited according to the availability of data, the regulatory authorities can test samples according to the needs of the early warning and fine-tune the model. Supervisors can use the forecast results of this model as a reference. If a company is found to be in crisis, it can focus on the company's financial situation in subsequent years.

## Figures and Tables

**Table 1:** Final early warning indicators.

Code	Indicators in the years of t-2 and t-3
X1	Current ratio
X2	Quick ratio
X3	Interest coverage ratio
X4	Asset-liability ratio
X5	Total asset growth rate (only significant in the years of t-2)
X6	Net profit growth rate (only significant in the years of t-2)
X7	Increase rate of business revenue
X8	Growth rate of owner's equity
X11	Current asset turnover
X12	Fixed asset turnover
X13	Total asset turnover
X14	Return on assets
X15	Return on equity
X16	Sales margin
X17	Ratio of profits to cost
X18	Earnings per share
X19	Net cash flow from operating activities per share
X20	Total cash recovery
X21	Cash flow coverage ratio
X22	Cash flow interest coverage ratio
X23	Ratio of net cash flow to net profit from operations
X25	Share ratio of the largest shareholder
X26	Share ratio of the top five shareholders

**Table 2:** Parameters of the logistic regression models in the year of *t*-2.

		B	S.E,	Wals	df	Sig.	Exp (B)	Exp (B) 95% C.I.	
								Lower limit	Upper limit
Steps 6f	F1	−1.265	0.357	12.581	1	0.000	0.282	0.140	0.568
	F2	−1.318	0.414	10.122	1	0.001	0.268	0.119	0.603
	F3	−2.678	0.605	19.591	1	0.000	0.069	0.021	0.225
	F4	−0.858	0.434	3.915	1	0.048	0.424	0.181	0.992
	F6	−1.349	0.578	5.443	1	0.020	0.260	0.084	0.806
	F7	−1.033	0.334	9.585	1	0.002	0.356	0.185	0.685
	Constant	0.120	0.313	0.147	1	0.702	1.127		

**Table 3:** Parameters of the logistic regression models in the year of t-3.

		B	S.E,	Wals	df	Sig.	Exp (B)	Exp (B) 95% C.I.	
								Lower limit	Upper limit
Steps 4d	F1	−2.509	0.515	23.741	1	0.000	0.081	0.030	0.223
	F4	18.025	4.419	16.641	1	0.000	67342482.46	11670.51	3886E.11
	F5	−1.145	0.457	6.274	1	0.012	0.318	0.130	0.780
	F9	−6.091	1.997	9.299	1	0.002	0.002	0.000	0.113
	Constant	0.781	0.391	3.988	1	0.046	2.183		

**Table 4:** Growth rate statistics.

Stock code	Data selection year	Sales growth rate (%)	Growth rate of owners' equity (%)	Value of growth rate of business income or owners' equity in each year before and after data selection (%)	Company status for data selection year
600228	2014	—	4.86	−1.38, 4.86, −0.11	A better financial position in 2014 led to the company's transition from ST to non-ST
600074	2014	—	2.78	1.67, 2.78, 0.94	A better financial position in 2015 led to the company's transition from ST to non-ST
000408	2013	4.79	—	−0.85, 4.79, −0.71	2013 is the year when the company achieved the transition from ST to non-ST
600234	2013	—	1.89	−0.44, 1.89, 0.12	A better financial position in 2013 led to the company's transition from ST to non-ST
002289	2013	—	3.11	−0.26, 3.11, 0.09	No changes took place in the company status this year
000068	2012	20.80	—	−0.93, 20.80, 5.88	A better financial position in 2012 led to the company's transition from ST to non-ST

**Table 5:** Model training results.

Years	Classification		Predicted number		Prediction accuracy (%)	Overall accuracy (%)
			Non-ST	ST		
Year of t-2	Actual number	Non-ST	52	9	85.2	87.7
		ST	6	55	90.2	

Year of t-3	Actual number	Non-ST	49	12	80.3	83.6
		ST	8	53	86.9	

**Table 6:** Model inspection results.

Years	Classification		Predicted number		Prediction accuracy (%)	Overall accuracy (%)
			Non-ST	ST		
Year of t-2	Actual number	Non-ST	35	6	85.4	87.8
		ST	4	37	90.2	

Year of t-3	Actual number	Non-ST	32	9	78	78
		ST	9	32	78	

**Table 7:** Classification rate of the training group.

Years	Classification		Predicted number		Prediction accuracy (%)	Overall accuracy (%)
			Non-ST	ST		
The year of t-2	Actual number	Non-ST	58	3	95.08	96.72
		ST	1	60	98.36	

The year of t-3	Actual number	Non-ST	59	2	96.72	96.72
		ST	2	59	96.72	

**Table 8:** Accuracy of the testing samples.

Years	Classification		Predicted number		Prediction accuracy (%)	Overall accuracy (%)
			Non-ST	ST		
The year of t-2	Actual number	Non-ST	36	5	87.8	92.68
		ST	1	40	97.56	

The year of t-3	Actual number	Non-ST	33	8	80.49	81.7
		ST	7	34	82.93	

**Table 9:** Comparison of prediction results of two early warning models in the year of t-2.

Analytical methods	Accuracy of training sample (%)			Accuracy of testing sample (%)		
	ST	Non-ST	Overall	ST	Non-ST	Overall
Logistic regression	90.2	85.2	87.7	90.2	85.4	87.8
BP neural network approach	98.36	95.08	96.72	97.56	87.8	92.68

**Table 10:** Comparison of prediction results of two early warning models in the year of t-3.

Analytical methods	Accuracy of training sample (%)			Accuracy of inspection sample (%)		
	ST	Non-ST	Overall	ST	Non-ST	Overall
Logistic regression	86.9	80.3	83.6	78	78	78
BP neural network approach	96.72	96.72	96.72	82.93	80.49	81.7

**Table 11:** Samples for the three models.

		Training set (60%)	Validation set (40%)	Test set
Model A	Company status	2016–2018	2016–2018	2019
	Financial data	2014–2016	2014–2016	2017

Model B	Company status	2015–2017	2015–2017	2019
	Financial data	2013–2015	2013–2015	2017

Model C	Company status	2016–2018	2016–2018	2019
	Financial data	2013–2015	2013–2015	2016

**Table 12:** Summary of model accuracy.

Category	Accuracy of training samples (%)	Accuracy of inspection samples (%)	Accuracy of test samples (%)
Model A	94.87	94.23	94.44
Model B	95.24	94.23	92.11
Model C	96.15	84.61	68.42

## Data Availability

The datasets used and/or analyzed during the current study are available from the corresponding author on reasonable request.
